# Effective Steroid Therapy for Reexpansion Pulmonary Edema

**DOI:** 10.31662/jmaj.2018-0038

**Published:** 2019-02-22

**Authors:** Katsuhiro Yoshikawa, Mami Miyata, Noriko Sueoka, Daigo Yamamoto

**Affiliations:** 1 Institutional affiliations: Kansai Medical University Medical Center, Osaka, Japan

**Keywords:** Reexpansion pulmonary edema, Pleural effusion, Steroid pulse therapy, Chest drainage

A 38-year-old woman was diagnosed with massive left pleural effusion caused by advanced breast cancer approximately 1 month ago (Figure 1A). A 12-French chest tube was placed, and 1000 mL of pleural effusion was drained for approximately 1 hour. No complications occurred. However, severe coughing and dyspnea developed upon completion of drainage. Coarse crackles were present in all left lung fields. Her peripheral blood oxygen saturation decreased to 93%. Computed tomography showed broad ground-glass opacities and poor expansion in the left lung, suggestive of reexpansion pulmonary edema (RPE) (Figure 1B). Methylprednisolone pulse therapy (1000 mg/day for 3 days) was administered. The symptoms improved the next day; after 4 days, computed tomography showed complete resolution of the radiological abnormalities (Figure 1C). RPE is rare (incidence, 0.2%) ^(1)^, but fatal (mortality rate, 20%) ^(2)^. Supportive therapy, such as positive pressure ventilation, is considered as the main treatment for RPE, but steroid pulse therapy also may be effective ^(3)^.

**Figure 1.**
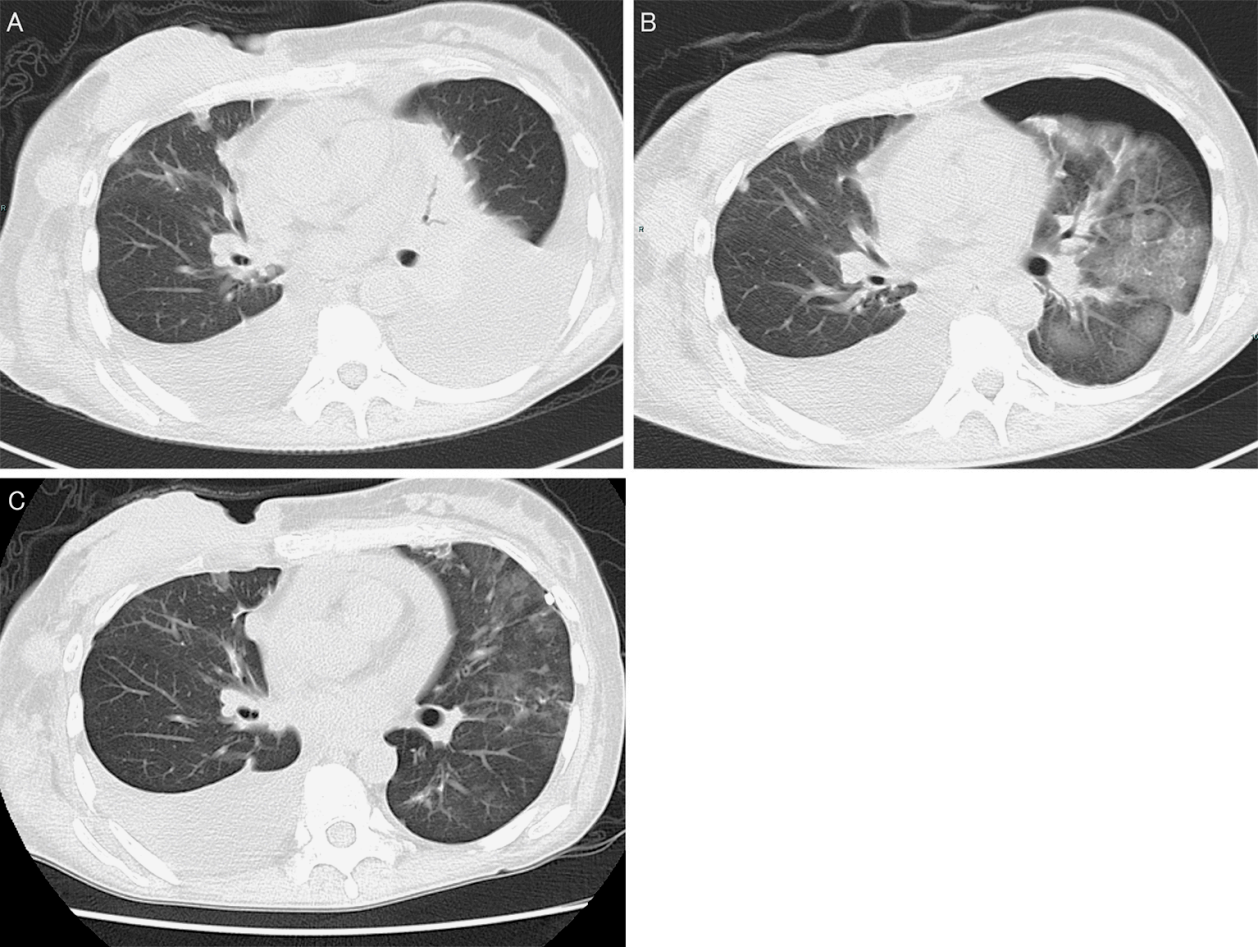
Computed tomography showing the onset of reexpansion pulmonary edema and improvement (A) Pre-drainage (B) Post-drainage (C) After 4 days.

## Article Information

### Conflicts of Interest

None

### Author Contributions

Katsuhiro Yoshikawa designed the study, and wrote the initial draft of the manuscript. All other authors have contributed to data collection and interpretation, and critically reviewed the manuscript. All authors approved the final version of the manuscript, and agree to be accountable for all aspects of the work with respect to ensuring that questions related to the accuracy or integrity of any part of the work are appropriately investigated and resolved.

### Approved by Institutional Review Board (IRB)

This study has been performed anonymously: therefore, approval by the IRB is not required based on Ethical Guidelines for Medical and Health Research Involving Human Subject by Ministry of Health, Labor and Welfare in Japan.

### Informed Consent

Written informed consent was obtained from the patient for publication of this “Image” and accompanying images.
